# Pilot Neuroimaging Evidence of Altered Resting Functional Connectivity of the Brain Associated with Poor Sleep After Acquired Brain Injury

**DOI:** 10.3390/jcm15020534

**Published:** 2026-01-09

**Authors:** Lai Gwen Chan, Jia Lin, Chin Leong Lim

**Affiliations:** 1 Tan Tock Seng Hospital, Singapore 308433, Singapore; jia.lin@nhghealth.com.sg; 2 Lee Kong Chian School of Medicine, Nanyang Technological University, Singapore 308232, Singapore; fabianlim@ntu.edu.sg; 3 College of Health Science, VinUniversity, Hanoi 12426, Vietnam

**Keywords:** brain injury, stroke, traumatic, sleep, resting state, functional connectivity

## Abstract

**Background/Objectives**: This study aimed to characterize objective sleep measures in subacute acquired brain injury (ABI) and examine if disturbed sleep is associated with poor recovery outcomes. Another objective was to compare the functional connectivity of the brain between ABI poor sleepers and ABI normal sleepers as measured by resting state functional magnetic resonance imaging (rs-fMRI). **Methods**: This was a pilot, prospective, observational study of ABI subjects compared with age and gender-matched healthy controls. A total of 27 ABI subjects (consisting of ischemic or haemorrhagic stroke, or traumatic injury) were recruited from the outpatient clinics of a tertiary hospital with a neurological centre, and 49 healthy controls were recruited by word-of-mouth referrals. Study procedure involved subjective and objective sleep measures, self-report psychological measures, cognitive tests, and structural and functional MRI of the brain. **Results**: The frequency of poor-quality sleep was 66.67% in the ABI group and not significantly different from 67.35% in the control group when compared by chi-squared test (*p* = 0.68). ABI subjects with poor sleep had worse performance on a test of sustained attention (Colour Trails Test 1) than healthy controls with poor sleep when compared by Student’s *t*-test (mean 55.95 s, SD ± 18.48 vs. mean 40.04 s, SD ± 14.31, *p* = 0.01). Anxious ABI subjects have poorer sleep efficiency and greater time spent awake after sleep onset (WASO). ABI-poor sleepers show significantly greater functional connectivity within a frontoparietal network and bilateral cerebellum. **Conclusions**: Sleep problems after ABI are associated with poorer cognitive and psychological outcomes. ABI-poor sleepers exhibit altered functional connectivity within regions that contribute to motor planning, attention, and self-referential processes, suggesting that disrupted sleep after ABI may impair the integration of sensorimotor and cognitive control systems, and therefore, impair recovery.

## 1. Introduction

Together, trauma and stroke are the top causes of acquired structural brain injuries (ABI) which often lead to chronic neurological disability [1]. Post-ABI sleep dysfunction is therefore of great importance due to the known restorative function of sleep [2] and the potential detrimental neurocognitive impact of sleep deprivation [3]. Moreover, Luo et al. showed through a systematic review that subjective poor sleep quality may affect as many as 66% of stroke patients [4], and a meta-analysis by Fulk et al. demonstrated that stroke survivors with a diagnosed sleep disorder suffer greater functional disabilities than those without [5]. Studies in populations with traumatic brain injuries have similarly shown that sleep disturbance is potentially associated with poorer rehabilitation outcomes such as the presence of agitation, cognitive impairments, and reduced quality of life [6,7]. Therefore, sleep is a potential therapeutic target for improving outcomes after ABI. To explore this potential, objective sleep measurements, relevant clinical outcome measures, and biomarkers of pathology and treatment response are needed. Neuroimaging may be one such biomarker.

Studies regarding structural neuroimaging changes in post-ABI sleep dysfunction have shown mixed results. Hence, more recent studies have used functional magnetic resonance imaging (fMRI), particularly of the resting state, to examine changes in functional connectivity of the brain. In a 2020 study among 28 individuals with mild traumatic brain injury, subjective poor sleep quality was associated with weaker resting state functional connectivity between the left parahippocampal gyrus and the precuneus, cerebellum, thalamus, and cingulate gyrus [8]. On the other hand, a similar resting state-fMRI study on 27 patients with insomnia after stroke showed abnormal local activities in multiple brain regions indicating over-arousal of the Default Mode Network (DMN) and over-sensitivity to audiovisual stimuli [9].

This study aimed to characterize objective sleep measures in subacute ABI and explore the relationship between poor sleep and neuropsychiatric outcomes. Another objective was to examine the functional connectivity of the brain as measured by resting state functional magnetic resonance imaging (rs-fMRI) through a subset of the ABI cohort.

## 2. Materials and Methods

This was a pilot, prospective, observational study of ABI subjects compared with age and gender-matched healthy controls that was approved by Domain Specific Review Boards of the National Healthcare Group. *A priori* sample size calculation for a study power of 0.8 was 38 subjects per group. ABI subjects were recruited from the outpatient clinics of a tertiary hospital with a neurological centre, and healthy controls were recruited by word-of-mouth referrals.

Subjects were eligible for the study if they were aged between 21 and 65 and spoke either English or Mandarin. ABI subjects were eligible if they had a documented diagnosis of traumatic brain injury of any severity or ischemia/haemorrhagic stroke and were not more than three months post-event by the time of baseline assessment. They also had to agree to stop taking sleep-inducing medications for seven days prior to study assessment for adequate washout of medication effects and to minimize potential confounding of results of the sleep tests. There were no specific dietary restrictions, and habitual caffeine consumption was allowed but only in the mornings and before 12 noon. This was to enhance subject acceptability of the study procedure.

Subjects were excluded if they had severe polytrauma, penetrating head injury, depressed skull fractures, neurosurgery breaching the dura mater, preinjury history of brain injury/intellectual disability/psychiatric disorder/substance abuse/sleep disorder/cognitive disorder, still in post-traumatic amnesia or on tracheostomy, any contraindication to MRI, or lacked a legally acceptable representative.

Medical records were reviewed to extract relevant clinical variables including demographics and length of hospital stay.

### 2.1. Outcome Measures

Subjective sleep measures used were a Sleep Diary, the Pittsburgh Sleep Quality Index (PSQI) [10], Epworth Sleepiness Scale (ESS) [11], and the Insomnia Severity Index (ISI) [12].

Objective sleep measures using unattended ambulatory overnight polysomnography for one night and wrist actigraphy for 7 consecutive days were performed only if a subject scored ≥ 5 on PSQI indicating poor sleep quality. One night of polysomnography was deemed sufficient since it was conducted in the subject’s home and was the best balance of subject acceptability of the study procedure with optimizing the likelihood of obtaining valid analysable results.

Neuropsychiatric outcome measures included anxiety and depression symptoms and cognitive function. The Hospital Anxiety and Depression Scale (HADS) [13] was used for the former, and cognitive functions were measured using the Montreal Cognitive Assessment (MoCA) [14], Colour Trails Test (CTT) [15], Symbol Digit Modalities Test (SDMT) [16], and the International Shopping List Task (ISLT) [17].

Structural and functional MRI was performed for a subset of recruited subjects who had both objective and subjective sleep measures, using a Siemens 3T Prisma scanner using a protocol which included T1 and T2 weighted sequences, Diffusion Tensor Imaging (DTI), Susceptibility Weighted Imaging (SWI), and 2 cycles of resting state fMRI for 10 min each.

### 2.2. Statistical Analysis

All analyses of non-MRI variables were performed using Statistical Package for Social Sciences (SPSS) version 25.0.

The first comparison was between ABI subjects and healthy controls, then between ABI subjects with poor sleep and ABI subjects with normal sleep. Categorical variables were compared using the chi-squared test and continuous variables using the Student’s *t*-test after satisfying the assumptions for parametric tests. The level of significance was set at <0.05.

For the neuroimaging dataset, we conducted a group-level independent component analysis (ICA) using MELODIC (Multivariate Exploratory Linear Optimized Decomposition into Independent Components), part of FMRIB’s Software Library (FSL) ver. 6.0.5, to investigate resting-state functional connectivity differences between ABI subjects with poor sleep and those with normal sleep. Preprocessed functional MRI data of the ABI subjects were temporally concatenated across subjects, and group-ICA was performed to identify common resting-state networks. Dual regression was then used to estimate subject-specific spatial maps and time series for each component, followed by voxel-wise non-parametric permutation testing (using FSL’s randomize) to assess group differences.

## 3. Results

A total of 27 ABI subjects and 49 healthy controls were recruited and underwent assessment. The ABI group consisted of 12 patients with neuroimaging evidence of traumatic injury to the brain, and 15 patients with stroke, of which 9 were ischemic (6 right-sided and 3 left-sided) and 6 were hemorrhagic. Of the hemorrhagic strokes, 5 were basal ganglia hemorrhages (4 on the right and 1 on the left), and 1 was a diffuse subarachnoid hemorrhage. The small sample sizes did not permit subgroup analysis by pathology. The frequency of poor-quality sleep was 66.67% in the ABI group and 67.35% in the control group (*p* = 0.68). On further testing with objective sleep tests, no differences were found in the sleep indices of both groups. However, ABI subjects with poor sleep had worse performance on a test of sustained attention (Colour Trails Test 1) than healthy controls with poor sleep (55.95 s SD ± 18.48 vs. 40.04 s ± 14.31, *p* = 0.01).

Next, within-group comparison was performed for the ABI group. Table 1 shows comparisons of neuropsychiatric measures which showed that those who slept poorly were significantly more anxious than those who slept normally (HADS-Anxiety mean score 4.0 ± SD 3.24 vs. 1.29 ± SD 1.49, *p* = 0.014).

Hence, Table 2 shows the comparisons of objective sleep indices between anxious ABI subjects with poor sleep and non-anxious ABI subjects with poor sleep using a cutoff score of 4 on HADS-Anxiety scale. Anxious ABI subjects have poorer sleep efficiency and greater time spent awake after sleep onset (WASO).

**Figure 1:**
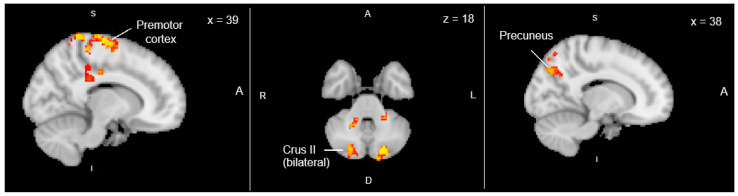
Differences in resting-state networks for poor sleep > normal sleep contrast in the ABI patients. Images were thresholded with a Threshold Free Cluster Enhancement-corrected (TFCE), *p* < 0.05.

Dual-regression analysis of the group ICA components revealed significantly greater functional connectivity in the ABI poor-sleep group compared with the ABI normal-sleep group. Cluster-level FWE (family-wise error rate)-corrected results showed increased connectivity within a frontoparietal network, with peak clusters localized to the right premotor cortex (x = 39), precuneus (x = 38), and bilateral cerebellar Crus II (z = 18) (Figure 1). These regions are consistent with cortico-cerebellar circuits implicated in sensorimotor planning and default-mode network activity. Our data showed no significant clusters survived correction in the opposite contrast (ABI normal-sleep > ABI poor-sleep).

## 4. Discussion

Our results are consistent with the idea that sleep problems after ABI are associated with poorer cognitive and psychological outcomes [18,19], despite a similar prevalence of poor sleep compared to non-ABI controls. This is likely due to a small sample size drawn from a population with a high baseline prevalence of poor sleep, resulting in an inadequately powered study.

This study demonstrates that individuals with ABI who report poorer sleep quality exhibit altered functional connectivity within cortico-cerebellar and frontoparietal networks, encompassing the premotor cortex, precuneus, and cerebellar Crus II. These regions contribute to motor planning, attention, and self-referential processes, suggesting that disrupted sleep after TBI may impair the integration of sensorimotor and cognitive control systems, and therefore impair recovery.

Disrupted sleep may lead to maladaptive changes or compensatory responses in task default mode network interactions [20], which could explain the increased precuneus connectivity and reduced attentional regulation we observed in poor sleepers. Greater connectivity in the premotor cortex and cerebellar Crus II also aligns with the role of sleep in motor recalibration and consolidation [21]. In ABI, sleep disruption may impair cerebellar contributions to predictive control, leading to compensatory hyperconnectivity and broader executive demands [22].

The generalizability of these findings is mainly limited by the heterogeneity of the sample in terms of the aetiology of the acquired injury, and the small sample size resulting in an underpowered study. However, this is the first study of its kind to our knowledge, and pilot studies such as this are vital to the generation of hypotheses for future research.

Nevertheless, to summarize, our study results suggest worse anxiety and sustained attention and altered functional brain connectivity measured at rest in ABI patients with poor sleep. This work calls for more attention to understanding the functional impact of sleep disturbances after ABI and searching for potential therapeutic targets and biomarkers of recovery and treatment response.

Future studies could explore potential distinguishing sleep and neuropsychological profiles in ABI of different aetiologies as well as their respective neuroimaging biomarkers on rs-fMRI. Interventional studies could potentially explore the efficacy of psychological or pharmacological treatments focused on anxiety in the treatment of sleep difficulties after ABI and use rs-fMRI signatures as biomarkers of treatment response.

## Figures and Tables

**Table 1:** Comparison of demographics and neuropsychiatric outcomes within ABI group (*n* = 27).

Baseline Variables	ABI Poor Sleep, PSQI ≥ 5 (*n* = 13)	ABI Normal Sleep, PSQI < 5 (*n* = 14)	*p*-Value
**Demographics**			
Age (mean, SD)	52.92 (±11.35)	53.21 (±15.16)	0.96
Male (*n*,%)	11 (84.6)	12 (85.7)	0.94
Chinese (*n*,%)	8 (61.5)	13 (92.9)	0.05
Employed (*n*,%)	10 (77.0)	8 (57.2)	0.65
Length of hospital stay (mean days)	18.70 (±15.72)	18.85 (±20.21)	0.98
**Psychological/Functional (mean, SD)**			
HADS-Anxiety	4.00 (±3.24)	1.29 (±1.49)	0.014 *
HADS-Depression	3.23 (±4.55)	0.86 (±1.03	0.09
HADS-Total	7.23 (±7.04)	2.14 (±2.18)	0.025 *
Functional Independence Measure (FIM)	124. 62 (±2.18)	124.71 (±4.53)	0.94
**Cognitive**			
Symbol Digit Modalities Test	48.08 (±8.88)	36.43 (±11.88)	0.008 *
Colour Trails 1 (sec)	55.95 (±18.48)	67.46 (±48.94)	0.42
Colour Trails 2 (sec)	97.37 (±26.54)	122.73 (±56.64)	0.15
Montreal Cognitive Assessment	26.83 (±1.72)	24.78 (±2.54)	0.08
ISLT Free recall	8.92 (±2.14)	7.93 (±2.95)	0.32
ISLT Weighted recall	58.54 (±15.70)	53.64 (±18.62)	0.47
ISLT Primacy	8.31 (±2.25)	7.79 (±3.14)	0.62
ISLT Recency	7.15 (±1.63)	6.07 (±2.64)	0.21
ISLT Intrusion	1.23 (±1.64)	0.86 (±1.10)	0.5
ISLT Repetition	2.23 (±2.31)	2.43 (±3.74)	0.87

* denotes a significant difference.

**Table 2:** Comparison of objective sleep indices between anxious and non-anxious ABI subjects with poor sleep.

Sleep Variable (Minutes)	ABI Poor Sleep and Anxious, PSQI ≥ 5 and HADS-A ≥ 4 (*n* = 5)	ABI Poor Sleep and Not Anxious, PSQI ≥ 5 and HADS-A < 4 (*n* = 3)	*p*-Value
**Sleep Diary (mean, SD)**			
Time in Bed (TIB)	446.67 ± 68.92	352.27 ± 196.79	0.62
Total Sleep Time (TST)	380.03 ± 43.44	321.92 ± 184.84	0.73
Sleep Onset Latency (SOL)	36.67 ± 36.55	15.82 ± 11.63	0.31
Sleep Efficiency (SE %)	85.70 ± 7.98	90.91 ± 1.68	0.23
**Polysomnograph (mean, SD)**			
TIB	372.30 ± 74.13	409.33 ± 1.61	0.33
TST	252.30 ± 125.71	182.83 ± 176.16	0.59
SOL	56.24 ± 72.89	7.61 ± 5.78	0.21
Wake After Sleep Onset (WASO)	59.40 ± 62.26	216.67 ± 178.49	0.26
SE (%)	65.47 ± 30.78	44.66 ± 42.95	0.51
**Actigraphy (mean, SD)**			
TIB	466.33 ± 109.14	421.31 ± 52.86	0.5
TST	375.05 ± 94.33	379.36 ± 49.09	0.94
SOL	19.00 ± 22.41	11.73 ± 0.12	0.51
WASO	61.92 ± 29.01	21.69 ± 3.91	0.04 *
SE (%)	79.57 ± 8.47	90.03 ± 0.33	0.05 *

* denotes a significant difference.

## Data Availability

Data sharing available upon reasonable request.

## Abbreviations

The following abbreviations are used in this manuscript:

ABI	Acquired brain injury
DMN	Default mode network
Rs-fMRI	Resting state functional magnetic resonance imaging
PSQI	Pittsburgh sleep quality index
ESS	Epworth sleepiness scale
ISI	Insomnia severity index
HADS	Hospital Anxiety and Depression Scale
MoCA	Montreal Cognitive Assessment
CTT	Colour Trails Test
SDMT	Symbol Digit Modalities Test
ISLT	International shopping list task
DTI	Diffusion tensor imaging
SWI	Susceptibility weighted imaging
ICA	Independent component analysis
MELODIC	Multivariate Exploratory Linear Optimized Decomposition into Independent Components
FSL	FMRIB’s Software Library
